# Street Noises and Health

**Published:** 1899-10

**Authors:** 


### STREET NOISES AND HEALTH.

WE HAVE on several occasions during the past seven or eight years in the columns of the JOURNAL, invited attention to the deleterious effects of noise upon the people, especially the sick. Many scientific papers have been read at medical societies on the subject, some of which have been published in this magazine. The popular magazines, too, have printed many able articles relating to and dealing with it in a manner that ought to command attention.

We are not aware that a single official in this city from the highest to the lowest has ever paid the slightest heed to the question, or taken any action looking toward the abatement of the nuisance. It is, however, a subject of growing consequence, increasing with the extension of the city and soon will attain such proportions as to compel action to abate it in part and to restrain it in other part.

There are many noises in a large city that are necessary to the conduct of its affairs, but there are many others quite unnecessary, made by the heedless, and tolerated by the indifferent public.

It is not our purpose at this time to particularise,—we have done that heretofore—but rather to again call up the subject in hopes, in view of the great exposition in prospect, that something may be done in the premises.

To emphasise the importance that attaches to the question, we subjoin a letter published in a recent edition of the New York *Tribune*, to which we invite thoughtful attention:

*To the Editor of the Tribune;*

Sir.—I was out when your reporter called yesterday, and I now ask a little of your space to emphasise the point of the value of specific complaints, full and frequent, in all cases of noise which is detrimental to health. Experts in London have at times shown that cases of insanity, softening of the brain and kindred ailments, even resulting in death, have been distinctly traced to persistent distressing noises on the street.

Headaches are often caused by the passing of wagons laden with iron and the unloading of coal. In England coal dealers are now required to deliver coal in bags, carried and emptied by hand.

When the Lexington Avenue road was being built, a friend of mine who was sick in Thirty-seventh street was made doubly ill by the noises day and night of unloading iron, etc., on that avenue.

Dr. Feeney, of the Health Board, made to your reporter what seems to me a very inadequate statement of the case. He knows, as I do, that there are two sections of the consolidation act which have been made a part of the Greater New York charter that confer extraordinary powers on the Board of Health, in the interests of the public even when purely sanitary questions seem not to be involved.

The Board has ample power, and if citizens will only send us the correct data we will see that the matter is brought directly before the proper authorities. This society did its share to suppress the dead man's curve at Fourteenth Street, but we no more than a grand jury indictment could prevail in the premises. When, however, the Board of Health sent out its mandate, the nuisance was abated, and this is but one of a dozen like instances.

The Board, I repeat, has ample and complete power, and all our citizens must remember this and fail not to report every case of unnecessary noise, with all the facts as to the name and date, to the Board direct, or to this society.

We want more "kickers." New-Yorkers are too long-suffering and complain too little. Our society has not one-half as much to do as it should have, if our people would be less pessimistic and have more confidence in their own judgment as to what is a nuisance, and what is "dangerous to life or detrimental to health."

Berlin and London are improving steadily in this matter of suppressing unnecessary street noises, and surely we should not lag as far behind.

JOSIAH C. PUMPELLY.
No. 12 East Twenty-third street, Sept. 8, 1899.
Secretary City Improvement Society.

That one city is making progress is shown by the following view of the question as it affects London. The *Medical Press and Circular* in speaking of the work of the London Society says:

The life of the average citizen is becoming more tolerable, so far as street noises are concerned, a happy state of things which is due not to the fact that he has become accustomed to such nuisances, but because the streets are quieter than they used to be. In a certain measure a good influence has been exerted in this direction by the London Society for the Suppression of Street Noises. Although only in the second year of its existence, the society, as its report for the past year shows, has been steadily pressing forward an admirable campaign against the soul-disturbing costermongers and newsboys, who make the thoroughfares hideous with their yells and howls. Moreover, it is satisfactory to note that the society has a well-designed program in the pursuit of which its members intend to expend their best energies. For example, among their objects of reform are included the compulsory fixing of India-rubber tires on all vehicles, floors of the same material for milk-carts, the establishment of kiosks in place of the screeching newspaper boys. But even if all these reforms were successfully attained, many more noises would still be left to be dealt with. The bawling coal hawker, for instance, is an intolerable nuisance, and the irritating, expressionless, metallic clang of the barrel organ should be banished altogether from the streets. Possibly, however, when the London Government Act comes into force, marked improvement will be noticed in regard to the prevalence of street noises, inasmuch as the new municipalities have been empowered to make by-laws for regulating and suppressing such nuisances, and there is every reason for supposing that full advantage will be taken of the clauses in the act relating thereto."

